# Patterns of Analgesic Prescribing and High‐Risk Prescribing in Primary Care in Ireland 2014–2022—A Repeated Cross‐Sectional Study

**DOI:** 10.1002/ejp.70115

**Published:** 2025-09-01

**Authors:** Molly Mattsson, Ahmed Hassan Ali, Fiona Boland, Michelle Flood, Ciara Kirke, Emma Wallace, Derek Corrigan, Mary E. Walsh, Tom Fahey, Brian MacKenna, Frank Moriarty

**Affiliations:** ^1^ School of Pharmacy and Biomolecular Sciences RCSI University of Medicine and Health Sciences Dublin Ireland; ^2^ Data Science Centre RCSI University of Medicine and Health Sciences Dublin Ireland; ^3^ National Medication Safety Programme HSE National Quality and Patient Safety Directorate Dublin Ireland; ^4^ Department of General Practice University College Cork Cork Ireland; ^5^ FutureNeuro RCSI University of Medicine and Health Sciences Dublin Ireland; ^6^ School of Public Health, Physiotherapy and Sports Science University College Dublin Ireland; ^7^ Department of General Practice RCSI University of Medicine and Health Sciences Dublin Ireland; ^8^ The Bennett Institute for Applied Data Science, Nuffield Department of Primary Care Health Sciences University of Oxford Oxford UK

## Abstract

**Background:**

Pain is a significant burden on individuals, healthcare systems and society. Analgesic drugs carry many therapeutic benefits; however, all drugs are associated with adverse effects and risk of harm. Non‐steroidal anti‐inflammatory drugs (NSAIDs) and opioids have been identified as particularly high‐risk due to the risk of side effects and/or dependency. This study aims to examine how patterns of analgesic prescribing have changed in primary care in Ireland between 2014 and 2022.

**Methods:**

Monthly data on medicines prescribed and dispensed in primary care on the means‐tested General Medical Services (GMS) scheme in Ireland was used. Prevalence, initiations, discontinuations, chronic use and high‐risk prescribing, as defined by Scottish Polypharmacy Guidance, were summarised per year.

**Results:**

The prevalence of overall analgesic use decreased slightly over time, with 48.3% of GMS‐eligible individuals dispensed an analgesic in 2014 and 46.3% in 2022. This was largely driven by decreasing NSAID use, from 29.4% in 2014 to 25.0% in 2022. Prevalence for all other analgesic drug classes increased; however, after age/sex adjustment, higher odds of use in 2022 versus 2014 only persisted for gabapentinoids and amitriptyline. Some forms of high‐risk prescribing increased over time, including NSAIDs dispensed with oral anticoagulants, corticosteroids and SSRIs, with fewer decreasing.

**Conclusion:**

There was an overall reduction of analgesic use in Ireland, driven by decreasing systemic NSAID use. Although most other analgesic drug classes are increasing, this may largely be explained by changing demographics, particularly the age profile of the population. Despite this, interventions addressing rising high‐risk prescribing may be needed.

**Statement of Significance:**

Analgesic drug classes are an important focus for improving medication safety. This study suggests analgesic use is falling in Ireland, particularly for systemic NSAIDs, especially in older adults where adverse effects may be most harmful. The increasing prevalence of other analgesics may largely be explained by an ageing population. Analgesic use, and high‐risk prescribing, remains high, suggesting a need for enhanced access to non‐pharmacological services and interventions and also improved education and deprescribing support for healthcare professionals to address high‐risk prescribing.

## 1 Background

Pain is a common reason for seeking medical care (St. Sauver et al. 2013), with prevalence of chronic pain estimated at 20%–44% (Dahlhamer et al. 2018; Fayaz et al. 2016; Yong et al. 2022). Pain management is complex, influenced by clinical and patient‐specific factors including diagnostic uncertainty, patient preferences, and challenges of physical and mental health multimorbidities (Skou et al. 2022; Wang and Doan 2024). Pharmacological management of pain offers many therapeutic benefits; however, all drugs have potential for adverse effects. Harm associated with acute pain treatment is more likely to be short‐term or reversible; however, prolonged analgesic use for chronic pain can pose various risks requiring careful consideration.

Among analgesic treatments, opioids are particularly high‐risk and are no longer recommended as a first‐line treatment for any form of chronic pain. In Ireland, they are not routinely recommended for most cases of acute pain except in in‐patient settings for short‐term use as part of balanced multimodal analgesia (National Clinical Programme for Anaesthesia 2022). Despite these guidelines, recent research has shown increases in opioid prescribing among individuals with public health cover, rising from 14.4% in 2010 to 16.3% in 2019, with the greatest increase in those ≥ 65 years (Norris et al. 2021). However, trends in opioid prescribing should be considered in the context of overall analgesic prescribing patterns. Changes may reflect variation in the prevalence of pain conditions and the balance between medication initiation, chronic use, and discontinuation. Decreased opioid use may lead to switching to alternative analgesics, each with their own risks. This includes non‐steroidal anti‐inflammatory drugs (NSAIDs), with risks of gastrointestinal bleeding, acute kidney injury, and adverse cardiovascular events, particularly in older adults (Davis and Robson 2016), and gabapentinoids, with risks of misuse and abuse (Driot et al. 2019) and increased mortality when used in combination with opioids (Bharat et al. 2024).

Reduction of medication‐related harm has become an increasing priority internationally, driven by the World Health Organisation's (WHO) Third Global Patient Safety Challenge, Medication Without Harm (WHO 2017). One of the challenge's three focus areas is higher‐risk situations, including the prescribing of specific medications like opioids and NSAIDs. Several other national/international initiatives to reduce medication harm with some focus on analgesics have been developed. Scotland's Polypharmacy Guidance highlights indicators of high‐risk prescribing, including multiple relating to NSAIDs, opioids and gabapentinoids (Scottish Government Polypharmacy Model of Care Group 2018), as do the Organisation for Economic Co‐operation and Development (OECD) quality indicators for prescribing in primary care (OECD 2017). In addition, more specific interventions to improve higher‐risk prescribing have been implemented, for example, the roll‐out of the UK PINCER intervention involved training pharmacists to provide feedback, education and support within GP practices, systematically identifying and proactively reviewing high‐risk prescribing, including NSAIDs (Avery et al. 2012).

Given increasing global emphasis on reducing medication‐related harm and well‐documented risks associated with analgesics, it is important to understand prescribing trends in different settings. Primary care plays a key role in pain management, accounting for the largest volume of analgesia prescribing. Therefore, this study aims to examine changes in analgesic prescribing in Irish primary care between 2014 and 2022.

## 2 Methods

This is a repeated cross‐sectional study of medicines dispensed in primary care in Ireland from 2014 to 2022. The protocol for the overarching project has been previously published (Mattsson et al. 2022). The study was approved by the RCSI University of Medicine and Health Sciences Research Ethics Committee (ref: REC202201015) and Health Service Executive (HSE) Reference Research Ethics Committee B (ref: RRECB1022FM).

### 2.1 Population and Data Sources

This study includes individuals eligible for the General Medical Services (GMS) scheme, which is based on age and income and covers approximately 32% of the population, typically those more socioeconomically deprived than the general population (Mattsson et al. 2023). Data on GMS prescribing is held by the Health Service Executive (HSE) Primary Care Reimbursement Service (PCRS), which processes pharmacy claims for dispensed medications. The PCRS pharmacy claims database is a national source widely used in pharmaceutical policy and pharmacoepidemiology research in Ireland (Sinnott et al. 2017).

Data for this study were obtained from the PCRS through an information request and provided to the research team under a data transfer agreement. Analgesics were identified using WHO Anatomical Therapeutic Chemical (ATC) Classification codes and include opioids, paracetamol, NSAIDs, topical analgesics, antimigraine preparations, gabapentinoids and low‐dose amitriptyline. See Table S1. Data were at the level of individual medications dispensed to a patient. For each item, information included anonymised identifiers for individuals, date of dispensing, sex and age group of the individual, drug ATC code, product name and strength, quantity dispensed and cost. Anonymised identifiers were created by the PCRS for the purpose of data sharing and cannot be linked to actual individuals by the researchers or others. Defined daily doses were applied to relevant ATC codes based on route of administration (Norweigan Institute of Public Health 2024).

### 2.2 Outcomes

Outcomes were derived to capture patterns of prescribed analgesia utilisation, including:

- Prevalence of use, both overall and by age group and sex
  ○ As supplementary analysis, volume of use measures by age group and sex
- Prevalence of initiations, discontinuations and chronic use
- Prevalence of high‐risk use (defined below).

All outcomes were calculated for each year from 2014 to 2022, except for initiations, discontinuations and chronic use, which required a run‐in period and so were calculated from 2015 onwards. Outcomes were derived for all drug classes. For opioids, outcomes were further calculated for each individual drug, as well as weak and strong opioids and long‐acting opioid formulations, as outlined in Table S1. Systemic NSAIDs outcomes were also derived for COX‐2 inhibitors and non‐selective NSAIDs, and among topical products for lidocaine plasters, capsaicin and topical NSAIDs. Products containing combinations of paracetamol and opioids were included in definitions for all outcomes relating to both paracetamol use and opioid use. Outcomes were also derived for other analgesics, specifically pregabalin, gabapentin and low‐dose amitriptyline.

Pattern of use outcomes derived include prevalence of use, initiations, discontinuations and chronic use, as well as high‐risk polypharmacy dispensing indicators as defined by the Scottish Polypharmacy Guidance (Scottish Government Polypharmacy Model of Care Group 2018). Prevalence, initiations, discontinuations, and chronic use were all further calculated by age group and sex. High‐risk dispensing indicators related to NSAIDs dispensed with medications increasing the risk of bleeding, acute kidney injury or seizures, opioids dispensed with medications increasing the risk of falls and delirium (outlined in Table S2), and opioids and gabapentinoids dispensed at doses increasing the risk of dependency (opioids > 90 mg OME/day, pregabalin > 800 mg/day, gabapentin > 4800 mg/day) (Scottish Government Polypharmacy Model of Care Group 2018). Prevalence was calculated as the proportion of individuals with any occurrence, and percentage of dispensings of the relevant analgesic drug class considered high risk. An overview of definitions for included outcomes is provided in Table S3.

For volume of use, number of dispensings, cost, and standard doses were expressed as rates per 1000 population by sex and age group. PCRS annual reports provide numbers of eligible individuals at the end of each calendar year (Primary Care Reimbursement Service (PCRS) 2023). Standard doses, accounting for prescription quantity, potency, pack size and strength, based on WHO Defined Daily Doses (DDD) (WHO Collaborating Centre for Drug Statistics Methodology 2018), were calculated for all medications. For opioids, oral morphine equivalents (OME) were also calculated. These were derived from both single and combination medications, with strengths mapped to standard dose for each component ingredient in the case of the latter (see Table S4).

In addition to describing outcomes over time, the aim of the analysis was to explore trends in prevalence and determine whether a linear trend was present over time. To examine the prevalence of individual drug classes and the percentage of people with a high‐risk dispensing, univariable and multivariable regression analyses were conducted, using a generalised linear model with a logit link and the binomial distribution. Univariable models included year as an independent variable, and multivariable models further adjusted for age group and sex. All analyses were conducted using Stata version 18 (College Station, TX: StataCorp LLC). Statistical significance was set at *p* < 0.05.

## 3 Results

**FIGURE 1:**
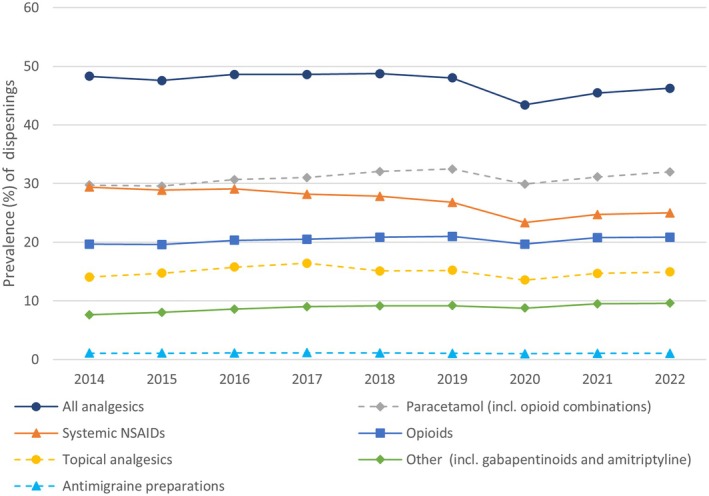
Prevalence of analgesic use between 2014 and 2022 by drug class.

The number of GMS eligible patients during the study period decreased from 1,768,700 in 2014 to 1,568,379 in 2022 (see Table S5). Trends over time are reported descriptively as changes for the full GMS population. The prevalence of all analgesic use decreased slightly during the study period, with 48.3% of all GMS eligible individuals dispensed an analgesic in 2014 and 46.3% in 2022. The largest decrease in prevalence for a drug class was systemic NSAIDs, from 29.4% in 2014 to 25.0% in 2022. Prevalence for all other analgesic drug classes increased during the study period; opioids increased from 19.7% in 2014 to 20.8% in 2022, paracetamol from 29.7% in 2014 to 32.0% in 2022, topical analgesics from 14.0% to 14.9% and other analgesics (low‐dose amitriptyline, pregabalin and gabapentin) from 7.6% in 2014 to 9.6% in 2022. For opioids, the individual drugs with the highest prevalence were codeine (which increased from 12.8% to 14.9%), tramadol (which decreased from 7.3% to 5.4%) and oxycodone (which increased from 1.2% to 2.1%), see Table S6. All drug classes saw a decrease in prevalence of use in 2020, coinciding with the onset of the COVID‐19 pandemic. Although prevalence increased again after 2020 for all drug classes, 2021 and 2022 prevalence were lower for all drug classes compared to 2019, with the exception of other analgesics (gabapentin, pregabalin and low‐dose amitriptyline). See Figure 1 and Table S6.

**FIGURE 2:**
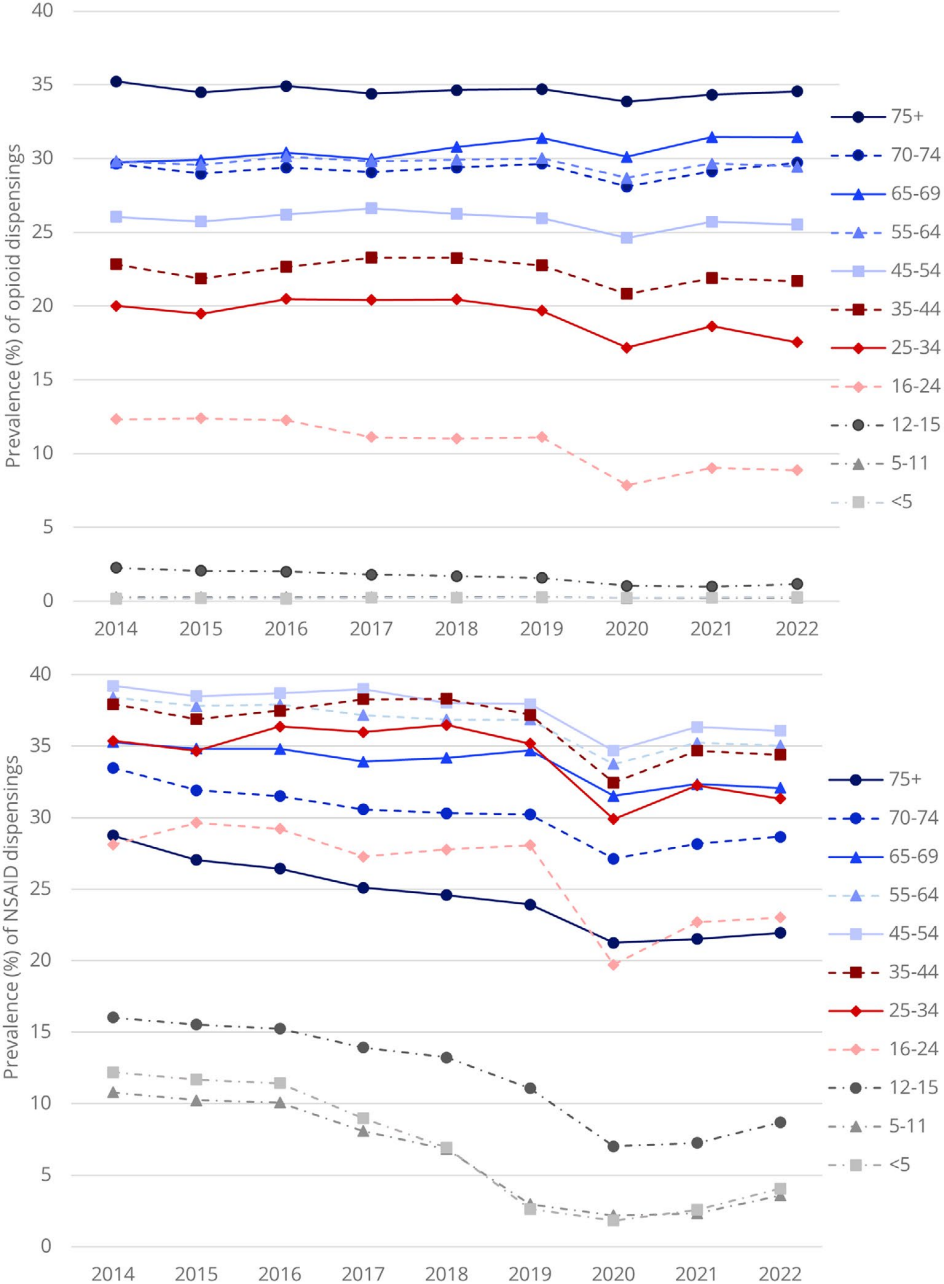
Prevalence of opioid (top) and systemic NSAID (bottom) use between 2014 and 2022 by age group.

For opioids, prevalence of use was consistently highest in the 75+ age group, remaining stable around 35%, followed by the 65–69, 55–64 and 70–74 age groups. Opioid use prevalence was stable throughout the study period for most age groups; however, decreases were seen for both 16–24 and 25–34 year olds and an increase was seen for 65–69 year olds. Similar results were seen for gabapentinoids, with the highest prevalence seen in the oldest age groups. For systemic NSAIDs, prevalence decreased for all age groups during the study. The highest prevalence was among middle‐aged adults, with the highest prevalences in those aged 45–54 years (39.2% in 2014 and 36.1% in 2022), followed by the 55–64 and 35–44 age groups. Comparatively lower prevalence was seen for the oldest age groups, with prevalence in those aged ≥ 75 decreasing from 28.8% to 22% (see Figure 2 and Table S7).

**FIGURE 3:**
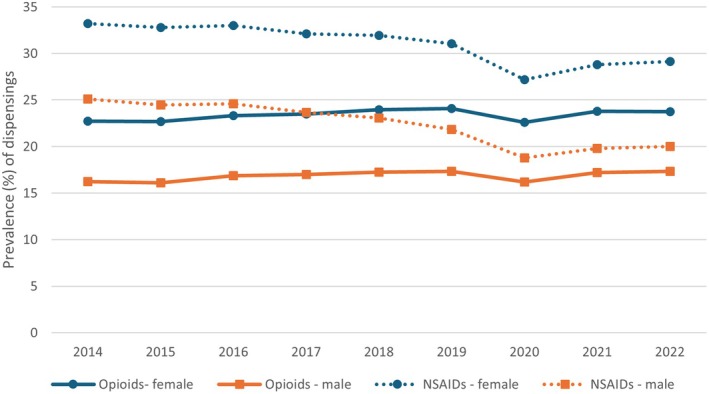
Prevalence of use of opioids (solid lines) and NSAIDs (dashed lines) for females (blue) and males (orange) between 2014 and 2022.

Females had a higher prevalence of use for all drug classes; for opioids this was 23%–24% during the study versus 16%–17% in males. Prevalence of systemic NSAID use decreased from 33% to 29% in women, and from 25% to 20% in men (see Figure 3 and Table S7). Rates of dispensings, cost, DDDs and OMEs calculated by age group and sex followed a similar pattern to the prevalence of use. Full results are available as additional data (see Data Availability Statement).

In regression models adjusting for age group and sex, the odds of being dispensed all drug classes decreased over the study, with the exception of other analgesics, that is, gabapentinoids and amitriptyline (see Table 1).

**TABLE 1:** Unadjusted and adjusted regression for the yearly change in prevalence of use (i.e., receipt of any dispensing) and high‐risk dispensings.

	Unadjusted OR	95% CI	Adjusted^a^ OR	95% CI
**Analgesic drug classes**				
Analgesics	0.98	0.98–0.98	0.98	0.98–0.98
Opioids	1.01	1.01–1.01	0.99	0.99–0.99
Systemic NSAIDs	0.96	0.96–0.96	0.96	0.96–0.96
Paracetamol	1.01	1.01–1.01	0.99	0.99–0.99
Others^b^	1.03	1.03–1.03	1.01	1.01–1.01
Topical analgesics	1.00	1.00–1.00	0.97	0.97–0.97
Antimigraine	0.99	0.99–0.99	0.99	0.99–0.99
**High‐risk medications/medication combinations**				
NSAID with antiplatelet	0.97	0.97–0.97	0.94	0.94–0.94
NSAID with anticoagulant	1.08	1.08–1.08	1.06	1.06–1.06
NSAID with corticosteroid	1.04	1.04–1.05	1.03	1.02–1.03
NSAID with SSRI	1.04	1.04–1.04	1.03	1.03–1.03
NSAID with ACE/ARB and diuretic	0.98	0.98–0.98	0.95	0.95–0.95
NSAID with ACE/ARB and metformin (over 65 s)	0.91	0.91–0.92	0.91	0.91–0.92
NSAID with lithium	0.99	0.98–1.00	0.98	0.97–0.98
Opioid with ≥ 2 other sedating or anticholinergic drugs (over 65 s)	0.98	0.98–0.99	0.98	0.98–0.98
High‐dose opioid^c^	1.02	1.02–1.03	1.00	1.00–1.00
High‐dose pregabalin^c^	1.01	1.01–1.01	1.00	1.00–1.01
High‐dose gabapentin^c^	1.04	1.03–1.05	1.02	1.01–1.03

^a^ Adjusted for age group and sex.

^b^ Pregabalin, gabapentin and low‐dose amitriptyline.

^c^ High dose use was defined based on the Scottish Polypharmacy Guidance as > 90 mg OME per day for opioids, > 800 mg per day for pregabalin and > 4800 mg per day for gabapentin.

Between 2015 and 2022, initiations of opioids (12.7% vs. 14.0%) and paracetamol (13.2% vs. 14.5%) increased slightly (Table 2). The prevalence of initiations dropped in 2020 for all drug classes and remained below 2019 levels in 2021 and 2022. Discontinuations remained relatively stable for all drug classes over the study period, whereas there were increases in the prevalence of chronic use of opioids (6.1% vs. 7.5%), paracetamol (5.0% vs. 7.7%) and other analgesics (4.7% and 6.2%). The percentage of all opioid dispensings constituting chronic use increased from approximately 30%–35%, whereas the percentage of chronic paracetamol dispensings increased from 29% to 38% of all paracetamol dispensings (see Table S6). Full results by age group and sex are available as additional data (see Data Availability Statement) and absolute numbers of individuals are reported in Table S8.

**TABLE 2:** Prevalence (%) of initiations, discontinuations, and chronic use of analgesic drug classes in the GMS population.^a^

	2015	2016	2017	2018	2019	2020	2021	2022
**Initiations**								
Opioids	12.7	13.7	14.0	14.2	14.3	13.2	13.9	14.0
Systemic NSAIDs	19.5	21.4	21.6	21.6	21.2	18.6	19.7	19.8
Paracetamol^b^	18.6	20.3	20.9	21.6	21.7	19.5	19.9	20.4
Antimigraine preparations	0.7	0.7	0.7	0.7	0.7	0.6	0.7	0.7
Topical analgesics	10.9	12.1	12.6	11.9	12.0	10.4	11.1	11.3
Other analgesics^c^	4.2	4.4	4.5	4.4	4.4	4.0	4.4	4.4
**Discontinuations**								
Opioids	10.5	11.3	11.6	11.7	11.8	10.7	11.3	11.5
Systemic NSAIDs	14.6	15.9	16.2	16.2	16.4	14.5	15.3	15.5
Paracetamol^b^	14.3	15.5	15.9	16.0	16.1	14.5	14.8	15.1
Antimigraine preparations	0.5	0.6	0.6	0.6	0.6	0.5	0.5	0.5
Topical analgesics	9.4	10.5	11.3	11.0	10.6	9.2	9.6	10.0
Other analgesics^c^	3.3	3.7	3.8	3.8	3.7	3.3	3.6	3.6
**Chronic use**								
Opioids	6.1	6.4	6.6	6.9	7.2	7.3	7.7	7.5
Systemic NSAIDs	3.6	3.5	3.5	3.5	3.5	3.6	3.8	3.6
Paracetamol^b^	8.5	9.0	9.4	10.1	10.6	11.2	12.1	12.2
Antimigraine preparations	0.3	0.3	0.3	0.4	0.3	0.3	0.3	0.4
Topical analgesics	2.9	3.3	3.5	2.8	3.0	3.3	3.8	3.8
Other analgesics^c^	4.7	5.1	5.4	5.6	5.8	5.7	6.1	6.2

^a^ Initiations and discontinuations are defined based on a medication‐free interval of 90 days before the first dispensing and after the last dispensing respectively, and chronic use for > 90 days.

^b^ Including opioid combinations.

^c^ Low‐dose amitriptyline, gabapentin and pregabalin.

**FIGURE 4:**
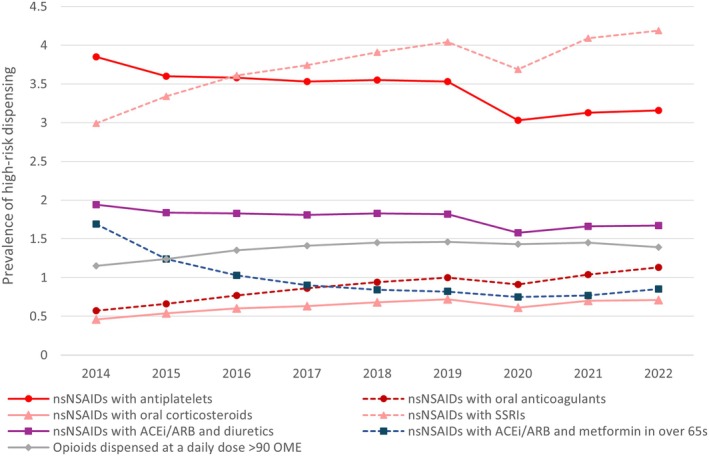
Prevalence of high‐risk dispensing** among users of the relevant analgesic drug class relating to bleeding risk (red), acute kidney injury risk (blue) and dependency risk (grey). *Indicators with prevalence < 0.4% or > 13% not shown. **High‐risk dispensing relates to all indicators of high‐risk use relating to analgesic drug classes listed in the Scottish Polypharmacy Guidance (Scottish Government Polypharmacy Model of Care Group 2018).

The prevalence of high‐risk dispensings varied during the study period. All high‐risk NSAID dispensings relating to the risk of bleeding increased, with the exception of non‐selective NSAIDs (dispensed with antiplatelets). The largest changes were seen for non‐selective NSAIDs dispensed with SSRIs and opioids dispensed with sedating agents in those aged ≥ 65 years. Among those receiving NSAIDs, the percentage also dispensed an SSRI increased from 3.0% to 4.2%. The percentage of those aged ≥ 65 years receiving an opioid dispensing with two or more sedating or anticholinergic drugs decreased from 15.4% in 2014 to 14.0% in 2022. Indicators relating to NSAIDs and the risk of acute kidney injury decreased during the study period. See Table S9 and Figure 4. Full results by age group and sex are available as additional data (see Data Availability Statement). In regression analysis, the change over time in the odds of having a high‐risk dispensing was similar after adjusting for age group and sex (Table 1).

## 4 Discussion

The prevalence of analgesic use in Ireland across most drug classes increased between 2014 and 2022, including opioids, gabapentinoids and paracetamol. However, this increase was offset by a decrease in NSAID use, and overall the prevalence of analgesia use decreased slightly. This is consistent with previous research on analgesia prescribing in Ireland (Moriarty et al. 2022; Norris et al. 2021), also showing higher rates of analgesia dispensings in Ireland than England (Mattsson et al. 2025). The results of this study indicate a higher prevalence of opioid use compared to Australia, France, Spain and Scandinavian countries (Chenaf et al. 2019; Hamina et al. 2022; Lalic et al. 2019; Xie et al. 2022). Additionally, the overall prevalence of analgesic use in Ireland appears higher than in Germany, the UK, and France (Jacob and Kostev 2018). However, the regression results, accounting for demographic changes in the population, indicate a lower chance over time of an individual being prescribed any analgesic class, with the exception of gabapentinoids and low‐dose amitriptyline. Importantly, the prevalence of several forms of high‐risk dispensing is rising, independent of demographic changes, particularly those relating to bleeding risk with NSAIDs.

All analgesic drug classes saw a decrease in prevalence of use in 2020, coinciding with the COVID‐19 pandemic. Although this increased since, 2021 and 2022 prevalence remained lower than in 2019 for all drug classes bar gabapentinoids and amitriptyline. Previous research in England found that COVID‐19 ‘lockdown’ measures did not impact opioid prescribing in primary care (Nawaf Sindi et al. 2022), with similar results in the Netherlands (Ellerbroek et al. 2024). Canadian research on the experience of patients with chronic pain during the pandemic suggested a negative impact on patients' experience of their care, with lack of access to prescribers/cancellation of medical appointments and increased medication prescribing in compensation for stopping physical/psychological treatments (Lacasse et al. 2021). A similar increase was not seen in Ireland, and the observed decrease in 2020 suggests the pandemic may have impacted access to prescribed analgesic drugs.

The prevalence of opioid, NSAID, and gabapentinoid use was persistently higher in women compared to men. It is well‐established that women have a higher chronic pain prevalence and are prescribed more analgesics, although the mechanisms behind this are not fully understood (Campbell et al. 2010; Mogil and Bailey 2010). The highest prevalence of opioid and gabapentinoid use was consistently seen in the oldest age groups. Middle‐aged groups had the highest prevalence of systemic NSAID use, with lower prevalence among older groups. NSAIDs in older adults have been identified as high‐risk prescribing, given renal and cardiovascular harms (Wongrakpanich et al. 2018) and the lower prevalence in these age groups is therefore a positive sign. The prevalence of chronic use of opioids, paracetamol, and other analgesics has risen. This is concerning given evidence of morbidity associated with long‐term opioid and gabapentinoid use, including sedation and dependence, and increasing numbers of deaths in which gabapentinoids have been implicated in Ireland and other countries (Durand et al. 2024; Kalk et al. 2022; Scottish Government Polypharmacy Model of Care Group 2018).

High‐risk opioid dispensing with two or more sedatives in those aged ≥ 65 years decreased during the study period, which may suggest a move away from inappropriate polypharmacy in this vulnerable age group. Rising prevalence of NSAIDs dispensed with an SSRI may be due to global increases in SSRI prescribing in recent years (Archer et al. 2022; Lalji et al. 2021; Luo et al. 2020), a trend which has also been seen in Ireland (McCool et al. 2022). Conversely, NSAIDs dispensed with acute kidney injury risk drugs decreased slightly. These have been targeted by recent interventions; the UK rollout of the pharmacist‐led PINCER intervention, targeting patients at risk of medication harm, across 343 general practices reduced hazardous prescribing by 16.7% at six months and 15.3% at 12 months postintervention (Rodgers et al. 2022). Similarly, the Data‐Driven Quality Improvement in Primary Care (DQIP) trial in Scotland found that professional education, informatics, and financial incentives to review patients' records to assess appropriateness significantly reduced targeted high‐risk prescribing (Dreischulte et al. 2016). The iSIMPATHY project introduced pharmacists in Scotland, Northern Ireland, and Ireland to conduct medication reviews (iSympathy Consortium 2023). In Ireland, 70.7% of the polypharmacy indicator occurrences identified were resolved post review (Kinahan et al. 2025), suggesting structured medicines review is an important strategy to consider for improving prescribing quality.

Ireland has historically had comparatively high rates of inappropriate prescribing, for example double the prevalence of potentially inappropriate prescribing among middle‐aged people versus Northern Ireland (Cooper et al. 2016). Recent research has shown an overall reduction in high‐risk prescribing in Ireland, though with significant variation between prescribers (Byrne et al. 2017). Greater availability of prescribing data in Ireland would enable audit and feedback interventions for quality improvement of prescribing in these areas. Previous educational interventions involving audit and feedback to improve the quality of prescribing had positive results in Ireland, that is, an initiative aimed at community prescribers saw an overall increase of 4.3% in prescribing of recommended (HSE 2020).

This study includes individuals eligible for the GMS scheme, which is means‐tested and covers approximately 32% of the population. Previous research has identified a higher prevalence of pain among older adults and individuals from disadvantaged backgrounds (Yong et al. 2022). A recent study on opioid prescribing in English primary care found a strong association of socioeconomic deprivation with opioid prescribing (Nowakowska et al. 2021). Deprivation is also linked to developing multimorbidity earlier in life and more rapid accumulation of physical and mental health conditions (Barnett et al. 2012), which, due to treatment complexity and single‐disease focus of clinical guidelines, may contribute to high‐risk prescribing and inappropriate polypharmacy (Jennings et al. 2024). In that context, it is therefore likely that Ireland's GMS population has a higher prevalence of analgesia use versus the general population.

Waiting times for GMS patients to access specialist care are extensive, resulting in patients with severe degeneration‐related chronic pain potentially waiting several years for joint replacement surgery, thus requiring strong analgesics in the meantime. In January 2025, over 61,000 individuals were awaiting an initial orthopaedic out‐patient appointment and nearly 12,000 an orthopaedic inpatient appointment (including joint replacement procedures), with around 7500 and 1000 people respectively waiting for more than 12 months (The National Treatment Purchase Fund 2024). Additionally, access to non‐pharmacological interventions, including exercise programmes, psychological therapies, acupuncture, and other treatments (e.g., transcutaneous electrical nerve stimulation) for GMS patients is often limited, despite some or all of these interventions being routinely recommended for multiple types of pain (e.g., osteoarthritis, rheumatoid arthritis, sciatica) (Allen et al. 2018; National Guideline 2016; Wood et al. 2023). For chronic primary pain (i.e., pain with no underlying condition adequately accounting for the pain or its impact), National Institute for Health and Care Excellence (NICE) guidelines recommend non‐pharmacological interventions as first‐line, with the use of opioids, gabapentinoids, paracetamol and NSAIDs all discouraged (NICE 2021). Although these recommendations have come under criticism for their restrictiveness (Eccleston et al. 2021), access to these non‐pharmacological interventions is vital.

The main strength of this study is the use of comprehensive data, which has high accuracy and completeness given its primary purpose for pharmacy reimbursement. Further, dispensing‐level data linked to individuals permitted examination of patterns of analgesic use. A limitation is the study only includes data relating to the means‐tested GMS eligible population, with the older, more disadvantaged population not fully representative of the general population. Secondly, the data lacks details of indication for the prescription and patients' diagnoses, so although drug classes examined are almost exclusively used for pain, we could not examine medication use by type of pain. Although all dispensings related to prescriptions issued in primary care, some of these medications may have been originally initiated in specialist settings. Previous evidence has noted that hospital‐initiated prescriptions can contribute significantly to chronic opioid use (Daunt et al. 2024). We also lacked data on analgesics obtained over‐the‐counter, and considering Ireland has one of the highest rates of use of non‐prescription medicines containing codeine (Richards et al. 2022), prevalence of opioid use may be underestimated. However, the provision of prescription medicines at low cost on the GMS scheme may reduce the likelihood of significant purchases of over‐the‐counter products. Finally, as we do not have access to eligibility start/end dates, or data on mortality, we are not eligible to account for these factors when analysing initiation and discontinuation patterns, and as data used relates to dispensings, we do not have information on individuals' adherence to dispensed medicines.

## 5 Conclusions

This study suggests an overall reduction of analgesic use in Ireland, driven by a decrease in use of systemic NSAIDs. Although prevalence for most other drug classes is rising, this may largely be explained by the increasingly older age profile of the population. Despite improvements in some high‐risk indicators, prescribing that increases bleeding risk and chronic use of opioids and gabapentinoids are increasing and remain a concern, suggesting a need for effective medicines review services, enhanced availability of non‐pharmacological interventions and measures to reduce waiting times for specialist care. It is important that further meaningful analgesic drug utilisation research is enabled through timely access to appropriate data to support policies and practices to ultimately improve medication appropriateness and patient safety.

## Author Contributions

F.M. conceived the study. All authors were involved in the design of the study. M.M. and F.M. collected and curated the data. M.M., F.M., and A.H.A. developed the analysis plan. M.M. and F.M. conducted the analysis of the data. All authors were involved in the interpretation of the analysis results. M.M. drafted the manuscript, and M.F., A.H.A., E.W., F.B., C.K., M.E.W., D.C., T.F., B.M., and F.M. critically revised the manuscript. F.M. acquired funding for the study.

## Conflicts of Interest

The authors declare no conflicts of interest.

## Supporting information


**Data S1:** ejp70115‐sup‐0001‐Supinfo01.docx.

## Data Availability

The datasets analysed during the current study are not publicly available as this was not covered by the Data Exchange Agreement between RCSI and HSE‐PCRS. Data can be requested from HSE‐PCRS via an information request as detailed at https://www.hse.ie/eng/staff/pcrs/pcrs‐publications/. Full results for initiations, discontinuations, chronic use, and high‐risk dispensings by age group and sex are available from Zenodo (www.doi.org/10.5281/zenodo.14870683).
